# A risk assessment model for colon cancer and precancerous lesions based on cancer screening and external validation of the NHANES database

**DOI:** 10.3389/fonc.2026.1751084

**Published:** 2026-04-13

**Authors:** Jing Wang, Ran Xiang, Chunhua He, Fuye Li

**Affiliations:** 1 Xinjiang Medical University, Urumqi, Xinjiang, China; 2 Physical Examination and Health Management Center, Affiliated Tumor Hospital of Xinjiang Medical University, Urumqi, Xinjiang, China

**Keywords:** cancer screening, colon cancer, NHANES database, precancerous lesions, risk assessment

## Abstract

**Objective:**

This study aimed to develop and externally validate a non-invasive predictive nomogram for stratifying individuals at elevated risk for colon cancer and precancerous lesions, with the goal of optimizing risk-stratified screening protocols. The model was derived from a clinical screening cohort and validated using the National Health and Nutrition Examination Survey (NHANES) database.

**Methods:**

The modeling cohort consisted of 400 participants who underwent colonoscopy, comprising 272 healthy controls and 128 patients histopathologically diagnosed with colon cancer or precancerous lesions. External validation was performed on 284 individuals from the NHANES database (191 healthy controls and 93 self-reported cases). All predictors were selected based on their non-invasive nature and clinical accessibility.

**Results:**

Significant intergroup differences were observed in age, body mass index (BMI), smoking history, alcohol consumption, and high-fat diet (all *P* < 0.05). Multivariable logistic regression confirmed age >55 years, BMI >25 kg/m², smoking, alcohol use, and a high-fat diet as independent risk factors for colonic neoplasia. The derived nomogram exhibited robust discriminative ability in both the modeling (AUC, 0.765) and validation (AUC, 0.761) cohorts. Decision curve analysis demonstrated that intervention guided by the nomogram yielded superior net clinical benefit at risk thresholds of >0.15 and >0.11 in the modeling and validation cohorts, respectively.

**Conclusion:**

This novel, non-invasive nomogram provides a reliable and pragmatic tool for individualized risk assessment of colon cancer and precancerous lesions. Its strong performance in both internal and external validations supports its potential utility in enhancing risk-stratified screening and early intervention strategies in diverse populations.

## 1 Introduction

Colon cancer remains one of the most commonly diagnosed malignancies worldwide, with persistently high incidence and mortality rates that pose a substantial threat to global health (1). Its pathogenesis is widely recognized as a multifactorial and multistep process, involving intricate interactions among genetic susceptibility, lifestyle factors, and environmental exposures (2). According to the World Health Organization (WHO) and the National Comprehensive Cancer Network (NCCN), colon precancerous lesions—including conventional adenomas, serrated polyps, and dysplasia associated with inflammatory bowel disease—serve as critical precursors in colorectal carcinogenesis (3). In contrast, early-stage colon cancer is defined as invasive carcinoma confined to the mucosa or submucosa (i.e., Tis or T1 stage), which is biologically and prognostically distinct from precancerous lesions (4).

Early detection and timely intervention of these lesions are essential to reducing the incidence and mortality of colon cancer (5). However, both colon cancer and its precursors often present with nonspecific or asymptomatic early manifestations, leading to delayed diagnosis and missed opportunities for optimal treatment in a considerable proportion of patients. Current screening modalities for colon cancer include fecal occult blood testing (FOBT) and colonoscopy, among others (6). Although colonoscopy remains the most accurate screening tool, its invasive nature, high cost, and requirement for extensive bowel preparation limit its feasibility for large-scale population-based screening (7, 8). Consequently, there is a growing need for non-invasive, cost-effective, and easily implementable risk stratification tools to identify high-risk individuals who would benefit most from colonoscopic evaluation (9, 10).

The nomogram, a widely used graphical predictive tool, offers a practical solution by visualizing the relationships between multiple clinical variables and quantifying their respective contributions to a given outcome, thereby providing clinicians with an intuitive and individualized risk assessment (11, 12). Recent large-scale cross-sectional studies have increasingly employed nomograms to integrate demographic, clinical, and lifestyle factors for colorectal cancer risk prediction, underscoring their emerging role in modern screening programs (13). The National Health and Nutrition Examination Survey (NHANES), a nationally representative epidemiological study of the U.S. population, collects comprehensive data on demographic characteristics, lifestyle behaviors, health status, and nutritional intake through standardized interviews and physical examinations, offering a valuable resource for developing and validating public health models (11, 14).

The primary outcome of the present study was the presence of colon cancer or its precancerous lesions. Accordingly, we aimed to develop a nomogram that estimates the individualized probability of these pathological conditions, thereby informing risk-stratified screening strategies and optimizing the allocation of endoscopic resources. It is worth noting that although colorectal cancer (CRC) is frequently evaluated as a single clinical entity, this study specifically focused on colon cancer and its precursors, excluding rectal lesions. This distinction is supported by evidence that colon and rectal cancers differ in embryonic origin, molecular pathways, and their association with lifestyle factors such as high-fat diet and metabolic syndrome. A colon-specific model may therefore provide more precise risk stratification and facilitate more targeted screening approaches.

## 2 Subjects and methods

### 2.1 Subjects

This study employed a retrospective cohort design. The modeling cohort (G1) consisted of 400 consecutive participants who underwent colonoscopy at the Digestive Endoscopy Center of our hospital between April 2021 and October 2024. Based on colonoscopic and histopathological findings, participants were classified into the healthy group (n = 272, individuals with no abnormal findings) and the disease group (n = 128, patients diagnosed with colon cancer or precancerous lesions).

For external validation, a separate cohort (G2) was derived from the National Health and NHANES database using the 2021–2023 data cycle. A total of 284 eligible individuals were identified through the Medical Conditions Questionnaire, specifically module MCQ560, which captures self-reported history of cancer. This validation cohort comprised 191 healthy controls and 93 cases with self-reported colon cancer or related conditions.

**Figure 1:**
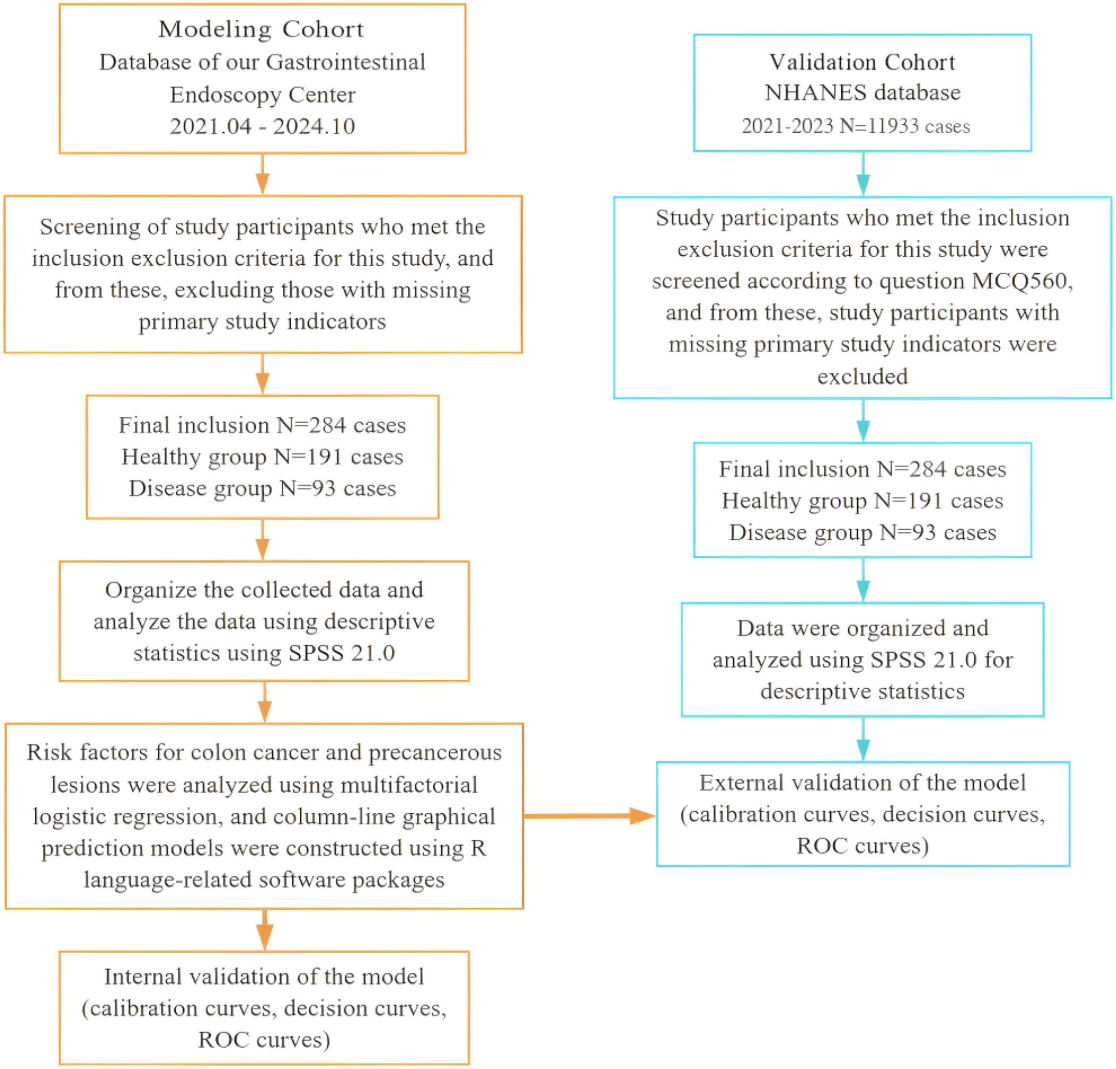
Study flowchart of participant selection and workflow for the modeling cohort (G1) and validation cohort (G2). This flowchart illustrates the systematic filtering of subjects from the Xinjiang clinical cohort (Modeling Group) and the NHANES database (Validation Group). The "Disease Group" includes pathologically or self-reported confirmed cases of colon cancer and precancerous lesions, while the "Healthy Group" comprises individuals with no such findings, following the exclusion of those with severe systemic diseases or incomplete records.

The selection of the 2021–2023 NHANES cycle was intentional, as it represents the most recent publicly available population-level data. This ensures that the model is validated against contemporary dietary patterns and lifestyle behaviors, which have undergone notable shifts in the post-pandemic era. The detailed participant selection process, along with the specific inclusion and exclusion criteria for both cohorts (detailed in Section 2.2), is illustrated in the study flowchart (**Figure 1**).

For the purpose of this analysis, time-zero was retrospectively defined as the date of colonoscopy for both cohorts. Accordingly, all participants were assumed to be free of a prior colorectal cancer diagnosis and asymptomatic at this reference point.

### 2.2 Inclusion and exclusion criteria

Inclusion criteria: ① Age > 18 years old; ② Polypoid lesions were detected by colonoscopy in the disease group, and the pathological diagnosis was colon cancer or precancerous lesions; ② Clear consciousness and able to communicate normally; ③ Complete clinical records were available, including documented demographic characteristics, lifestyle factors, and required laboratory test results. Exclusion criteria: ① Confusion of consciousness and unable to communicate normally; ② Accompanied by severe systemic diseases, defined as New York Heart Association (NYHA) class III/IV heart failure, severe chronic obstructive pulmonary disease (COPD), or end-stage renal disease; ③ Presence of other malignant tumors within the past 5 years, primary or secondary immunodeficiency, or clinically significant coagulation disorders (e.g., International Normalized Ratio [INR] > 1.5 without anticoagulation). All research procedures have been reviewed and approved by the Ethics Committee of our hospital.

### 2.3 Colonoscopy and pathological examination

Standard colonoscopy was performed by experienced endoscopists following routine bowel preparation. All detected lesions were evaluated and retrieved via biopsy or endoscopic resection (EMR/ESD). Specimens were fixed in 10% formalin, paraffin-embedded, and processed for hematoxylin-eosin (HE) staining. Pathological diagnosis was confirmed by independent pathologists according to standardized classification.

### 2.4 Clinical data collection

The collected clinical data were categorized into four domains: (1) Demographics: age, sex, and education level; (2) Anthropometric and lifestyle factors: BMI, smoking history, alcohol consumption, and dietary habits. These predictors were labeled as non-invasive indicators because they are acquired exclusively through patient interviews and simple physical measurements, avoiding the need for invasive procedures, specialized imaging, or expensive molecular assays; (3) Medical history: history of gallbladder surgery, hypertension, and diabetes; and (4) Laboratory indicators: total cholesterol, hemoglobin, platelet count, and fasting blood glucose. To ensure clinical applicability, continuous variables (age and BMI) were transformed into binary categorical variables using established thresholds: age > 55 years and BMI > 25 kg/m². For dietary factors, a ‘high-fat diet’ was defined as an average daily fat intake exceeding 30% of total caloric intake, while a ‘high-fiber diet’ was defined as a daily intake of ≥25g of dietary fiber (15, 16), assessed via standardized food frequency questionnaires. Missing data proportions were <5% per variable and had been managed using complete-case analysis. All variables were assessed for multicollinearity using Variance Inflation Factors (VIFs), and no multicollinearity was observed (VIF < 5 across all predictors).

### 2.5 Definition of colon cancer and precancerous lesions

This study focused exclusively on colonic lesions, excluding rectal cancer and rectal precancerous lesions, to ensure the homogeneity of the disease group. This distinction is based on the evidence that colon and rectal cancers exhibit significant differences in embryonic origin, anatomical structure, and biological response to lifestyle factors such as high-fat diets and metabolic syndrome.

Colon precancerous lesions refer to histopathologically confirmed abnormalities that are strongly associated with an increased risk of malignant transformation to colon cancer. These lesions primarily include conventional adenomas (e.g., tubular, tubulovillous, and villous adenomas), serrated adenomas (including sessile serrated lesions and traditional serrated adenomas), adenomatosis conditions (such as familial adenomatous polyposis and sporadic adenomatous polyposis), dysplasia arising from inflammatory bowel disease, and dysplastic aberrant crypt foci. These entities are recognized as key intermediates in the colorectal carcinogenesis pathway.

Early colon cancer is defined as a colonic epithelial neoplasm in which malignant cells invade the lamina propria or submucosa but do not penetrate through the muscularis propria into deeper layers. This corresponds to pathological Tis (carcinoma *in situ*) or T1 stage according to the American Joint Committee on Cancer (AJCC) staging system, regardless of tumor size or the presence of lymph node metastasis.

### 2.6 Statistical analysis

Statistical analyses were performed using SPSS and R software. Continuous variables were presented as mean ± standard deviation (SD) and compared between groups using the independent samples t-test. Categorical variables were expressed as frequencies and percentages (n, %) and compared using the chi-square (χ²) test.

In the modeling process, all independent predictors—including initially continuous variables such as age and BMI—were transformed into categorical variables based on clinically established thresholds to facilitate the construction of a point-based nomogram scoring system. While this dichotomization enhances clinical applicability, we acknowledge that it may result in some loss of predictive information and may obscure potential non-linear relationships compared to using continuous variables.

Predictor selection was guided by both statistical significance in univariate analyses and clinical relevance supported by the literature (17). Interaction terms among predictors were intentionally omitted to maintain model parsimony and ensure that the resulting point-based scoring system remained intuitive and easily applicable in rapid clinical assessments. Although family history—a well-established risk factor—was unavailable in the modeling cohort, its potential influence was indirectly addressed through sensitivity and subgroup analyses. Specifically, sex was included in a sensitivity analysis to assess model stability, and subgroup analyses stratified by demographic and lifestyle factors were conducted to evaluate the consistency and robustness of the remaining predictors across different populations.

To ensure model stability and minimize the risk of overfitting, sample size adequacy was assessed using the Events Per Variable (EPV) criterion. A minimum of 10–20 events per predictor is generally recommended for reliable prediction model development. With 128 events in the modeling cohort and five included predictors (age, BMI, smoking history, alcohol consumption, and high-fat diet), the EPV reached 25.6, substantially exceeding the methodological requirement. In the validation cohort, 93 events yielded an EPV of 18.6, which remains within the acceptable range and supports the statistical reliability of the external validation.

A nomogram was constructed using the rms package in R based on the independent predictors identified in the multivariable logistic regression model. Model performance was evaluated in terms of discrimination and calibration. Discriminative ability was assessed using the concordance index (C-index) and the area under the receiver operating characteristic curve (AUC). Calibration was evaluated through calibration plots (including calibration slope and intercept) and the Hosmer–Lemeshow goodness-of-fit test. In addition, the Brier score, net reclassification improvement (NRI), and integrated discrimination improvement (IDI) were calculated, with 95% confidence intervals estimated using the PredictABEL package in R.

Optimal risk thresholds for clinical decision-making were determined using Youden’s index and decision curve analysis (DCA). In the modeling cohort, a risk threshold of 0.15 was identified as providing the greatest net clinical benefit, while a threshold of 0.11 was derived for the validation cohort. To assess the stability of these thresholds, sensitivity analyses were conducted across a range of probability cut-offs (0.10–0.20), confirming that the selected points yielded robust and consistent net reclassification improvement. A two-tailed p-value < 0.05 was considered statistically significant throughout all analyses.

## 3 Results

### 3.1 Baseline data comparison

Baseline demographic and clinical characteristics of the modeling cohort are summarized in **Table 1**. Compared to the healthy group, the disease group exhibited significantly higher proportions of individuals aged >55 years, with BMI >25 kg/m², and with a history of smoking, alcohol consumption, and high-fat diet (all *P* < 0.05). No statistically significant differences were observed between the two groups regarding gender, education level, history of gallbladder surgery, hypertension, diabetes, high-fiber diet, or laboratory parameters including total cholesterol, hemoglobin, platelet count, and fasting blood glucose (all *P* > 0.05).

**Table 1:** Baseline characteristics of the modeling cohort (G1) (categorical variables: n (%); continuous variables: mean ± SD).

Variables	Disease group (*n* = 128)	Healthy group (*n* = 272)	*t/χ* ^2^	*P*
Categorical variables, n (%)				
Age (years)			24.190	<0.001
>55	62 (48.44)	65 (23.90)		
≤ 55	66 (51.56)	207 (76.10)		
Gender			0.722	0.395
Male	81 (63.28)	160 (58.82)		
Female	47 (36.72)	112 (41.18)		
BMI (kg/m^2^)			11.493	0.001
>25	88 (68.75)	138 (50.74)		
≤ 25	40 (31.25)	134 (49.26)		
Cultural level			0.707	0.400
High school and above	86 (67.19)	171 (62.87)		
Junior high school and below	42 (32.81)	101 (37.13)		
History of gallbladder surgery			0.289	0.591
Yes	42 (32.81)	82 (30.15)		
No	86 (67.19)	190 (69.85)		
Smoking history			9.564	0.002
Yes	68 (53.13)	100 (36.76)		
No	60 (46.87)	17 2 (63. 24)		
Drinking history			17.503	<0.001
Yes	71 (55.47)	91 (33.46)		
No	57 (44.53)	181 (66.54)		
History of high blood pressure			0.532	0.466
Yes	69 (53.91)	136 (50.00)		
No	59 (46.09)	136 (50.00)		
History of diabetes			0.322	0.571
Yes	47 (36.72)	92 (33.82)		
No	81 (63.28)	180 (66.18)		
High-fiber diet			0.171	0.679
Yes	56 (43.75)	125 (45.96)		
No	72 (56.25)	147 (54.04)		
High-fat diet			16.274	<0.001
Yes	72 (56.25)	95 (34.93)		
No	56 (43.75)	177 (65.07)		
Continuous variables, mean ± SD				
Total cholesterol (mmol/L)	4.90 ± 0.80	5.03 ± 0.89	1.417	0.157
Hemoglobin (g/L)	147.00 ± 14.00	148.00 ± 18.00	0.609	0.543
Platelet count (× 10^9^/L)	226.00 ± 51.00	229.00 ± 45.00	0.595	0.552
Fasting blood glucose (mmol/L)	5.00 ± 0.90	5.17 ± 1.02	1.584	0.114

Within the disease group of the modeling cohort (n = 128), the distribution of pathologically confirmed lesions—including conventional adenomas, serrated polyps, and early-stage colon cancer—was consistent with the diagnostic criteria defined in Section 2.5, thereby ensuring a representative spectrum of precancerous and early malignant colonic lesions.

### 3.2 Multivariate logistic regression analysis

The five variables that demonstrated statistical significance in the univariate analysis (**Table 2**) were subsequently entered into a multivariable logistic regression model to assess their independent effects. The results of the multivariable analysis identified age >55 years, BMI >25 kg/m², smoking history, alcohol consumption, and high-fat diet as independent predictors of colon cancer and precancerous lesions (all *P* < 0.05). The detailed classification criteria and categorical assignments for each predictor included in the model are presented in **Table 3**, ensuring that all variables were analyzed as discrete entities during regression modeling. The complete results of the multivariable logistic regression analysis, including regression coefficients (β), standard errors (SE), Wald statistics, odds ratios (OR), and corresponding 95% confidence intervals (CI), are provided in **Table 4**.

**Table 2:** Baseline characteristics of the validation cohort (G2) (categorical variables: n (%); continuous variables: mean ± SD).

Variables	Disease group (*n* = 93)	Healthy group (*n* = 191)	*t/χ* ^2^	*P*
Categorical variables, n (%)				
Age (years)			15.842	<0.001
>55	43 (46.24)	44 (23.04)		
≤ 55	50 (53.76)	147 (76.96)		
Gender			1.111	0.292
Male	62 (66.67)	115 (60.21)		
Female	31 (33.33)	76 (39.79)		
BMI (kg/m ^2^ )			6.599	0.010
>25	65 (69.89)	103 (53.93)		
≤ 25	28 (30.11)	88 (46.07)		
Cultural level			1.243	0.265
High school and above	67 (72.04)	125 (65.45)		
Junior high school and below	26 (27.96)	66 (34.55)		
History of gallbladder surgery			0.160	0.689
Yes	28 (30.11)	62 (32.46)		
No	65 (69.89)	129 (67.54)		
Smoking history			8.495	0.004
Yes	50 (53.76)	68 (35.60)		
No	43 (46.24)	123 (64.40)		
Drinking history			13.837	<0.001
Yes	50 (53.76)	59 (30.89)		
No	43 (46.24)	132 (69.11)		
History of high blood pressure			0.389	0.533
Yes	46 (49.46)	102 (53.40)		
No	47 (50.54)	89 (46.60)		
History of diabetes			0.057	0.811
Yes	32 (34.41)	63 (32.98)		
No	61 (65.59)	128 (67.02)		
High-fiber diet			0.062	0.804
Yes	38 (40.86)	81 (42.41)		
No	55 (59.14)	110 (57.59)		
High-fat diet			5.729	0.017
Yes	51 (54.84)	76 (39.79)		
No	42 (45.16)	115 (60.21)		
Continuous variables, mean ± SD				
Total cholesterol (mmol/L)	4.88 ± 0.75	5.05 ± 0.88	1.635	0.103
Hemoglobin (g/L)	146.48 ± 13.66	147.07 ± 18.37	0.299	0.765
Platelet count (× 10 ^9^ /L)	223.20 ± 52.77	228.91 ± 44.65	0.952	0.342
Fasting blood glucose (mmol/L)	5.02 ± 0.91	5.26 ± 1.05	1.909	0.057

**Table 3:** Multi-factor analysis assignment table.

Indexes	Variable	Assignment
Colon cancer and precancerous lesions	Dependent Variable	Yes=1, No=0
Age	Independent Variable	">55"=1, " ≤55 "=0
BMI	Independent Variable	">25"=1, " ≤25 "=0
Smoking history	Independent Variable	Yes = 1, No = 0
Drinking history	Independent Variable	Yes = 1, No = 0
High-fat diet	Independent Variable	Yes = 1, No = 0

**Table 4:** Multivariate logistic regression analysis.

Variables	*β*	*SE*	*Wald*	*OR*	*P* -value	95% *CI*
Age	1.212	0.248	23.882	3.361	<0.001	2.0 67 -5.4 66
BMI	0.837	0.248	11.408	2.310	0.001	1.4 21 -3.7 54
Smoking history	0.655	0.239	7.530	1.924	0.006	1.2 06 -3. 071
Drinking history	0.887	0.239	13.805	2.428	<0.001	1.52 1 -3.8 77
High-fat diet	0.918	0.239	14.747	2.505	<0.001	1.5 68 -4.0 04

### 3.3 Construction of a nomogram for predicting colon cancer and precancerous lesions

**Figure 2:**
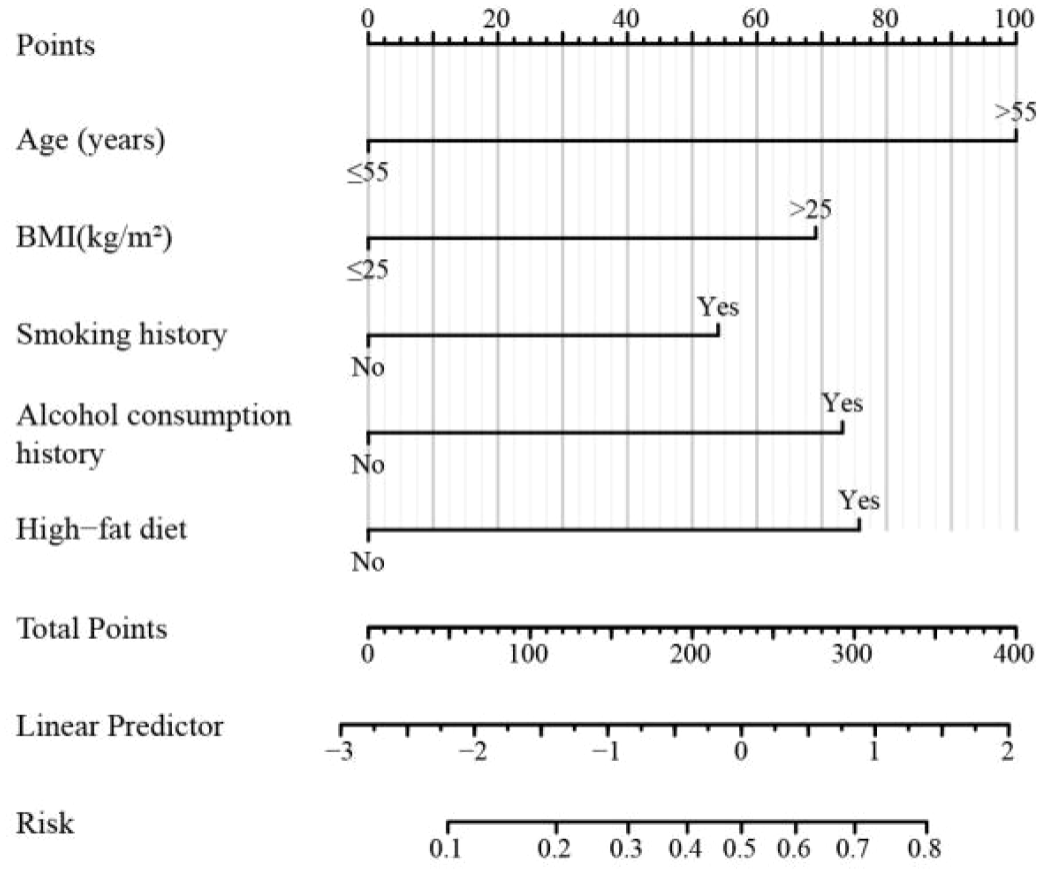
Nomogram for predicting the risk of colon cancer and precancerous lesions. This nomogram integrates age, BMI, smoking history, alcohol consumption, and high-fat diet to estimate individual risk scores; the total points correspond to a specific predicted risk probability for clinical stratification.

Based on the results of the multivariable logistic regression analysis, a predictive nomogram was constructed to estimate the risk of colon cancer and precancerous lesions. The model incorporated five independent predictors: age, BMI, smoking history, alcohol consumption, and high-fat diet. The nomogram is presented in **Figure 2**.

### 3.4 Internal validation

**Figure 3:**
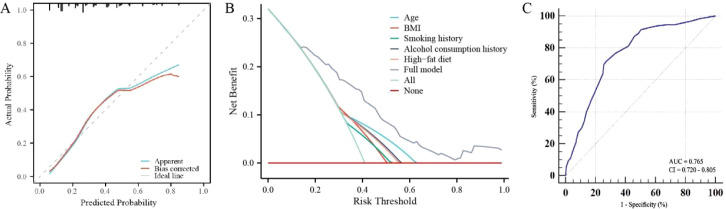
Internal validation of the nomogram model for the modeling cohort (G1). **(A)** Calibration curve; **(B)** Decision Curve Analysis (DCA); **(C)** ROC curve.

The calibration curve for the modeling cohort (G1) demonstrated good agreement between predicted probabilities and observed outcomes, with a C-index of 0.763 (95% CI: 0.714–0.813) for colon cancer and precancerous lesions (**Figure 3A**). Decision curve analysis (**Figure 3B**) revealed that when interventions were guided by the nomogram’s predictions, a risk threshold exceeding 0.15 provided greater net clinical benefit compared to either the “treat-all” or “treat-none” strategies. ROC curve analysis yielded an AUC of 0.765, indicating satisfactory discriminative ability (**Figure 3C**).

The model’s predictive accuracy was further supported by a Brier score of 0.182. Additionally, the net reclassification improvement (NRI) was 0.352 (95% CI: 0.124–0.580) and the IDI was 0.085 (*P* < 0.001), both indicating significant enhancement in reclassification ability compared to baseline demographic factors alone (all *P* < 0.05). These findings confirm that the incorporation of lifestyle indicators substantially improves the model’s predictive performance.

**Figure 4:**
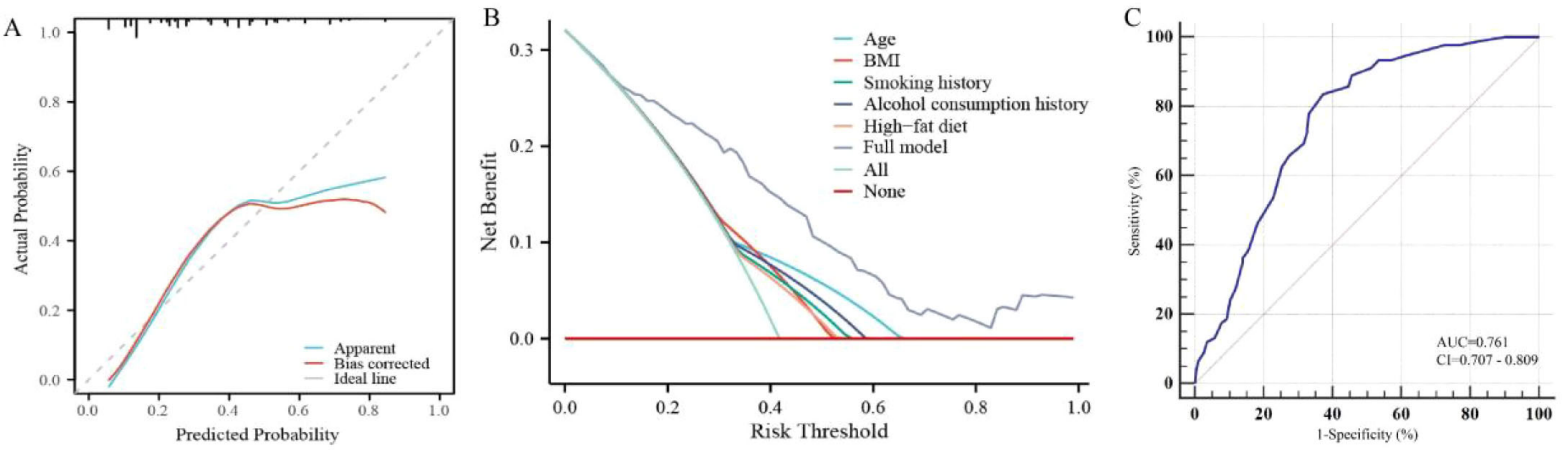
External validation of the nomogram model for the validation cohort (G2). **(A)** Calibration curve; **(B)** Decision Curve Analysis (DCA); **(C)** ROC curve. The ROC curves display the diagnostic performance of the nomogram in the modeling and validation groups. The area under the curve (AUC) values of 0.765 and 0.761, respectively, indicate good discriminative ability of the model in identifying high-risk individuals.

The Hosmer–Lemeshow goodness-of-fit test yielded a non-significant result (*χ*² = 14.03, *P* = 0.081), indicating no evidence of poor calibration in the external validation cohort. Consistent with the graphical calibration assessment presented in **Figure 4A**, the model maintained robust calibration across the risk spectrum. The calibration slope and intercept further corroborated the model’s predictive accuracy, despite the inherent heterogeneity between the development and validation populations.

### 3.5 External validation

Despite substantial geographical and genetic differences between the Xinjiang clinical cohort and the U.S. NHANES population, a comparative analysis of the primary predictors—specifically the proportions of individuals aged >55 years, with BMI >25 kg/m², and consuming a high-fat diet—revealed no statistically significant differences between the disease groups of the modeling and validation cohorts (all *P* > 0.05, see **Tables 1**, **2**). This comparability provided a statistical basis for cross-cohort application of the model.

In the validation cohort (G2), the calibration curve demonstrated good agreement between predicted probabilities and observed outcomes, with a C-index of 0.761 (95% CI: 0.705–0.818) for colon cancer and precancerous lesions (**Figure 4A**). Decision curve analysis (**Figure 4B**) indicated that intervention guided by the nomogram’s predictions yielded greater net clinical benefit when the risk threshold exceeded 0.11. ROC curve analysis yielded an AUC of 0.761, confirming the model’s sustained discriminative ability in an independent population (**Figure 4C**). The Brier score was 0.191, and both the net reclassification improvement (NRI = 0.284, *P* = 0.012) and IDI remained statistically significant, reinforcing the model’s robust performance in the external validation cohort.

The Hosmer–Lemeshow goodness-of-fit test yielded a non-significant result (*χ*² = 15.27, *P* = 0.054), indicating acceptable calibration. Given that this test is sensitive to sample size, greater emphasis was placed on the graphical calibration curve (**Figure 4A**), which demonstrated a high degree of concordance between predicted and observed outcomes, further supported by the calibration slope and intercept.

Regarding model transportability, discriminative performance remained stable across cohorts. However, a slight shift in the calibration intercept (calibration-in-the-large) was observed during external validation, reflecting differences in baseline risk between the Chinese and U.S. populations. Accordingly, risk thresholds were adjusted to 0.15 and 0.11 for the modeling and validation cohorts, respectively, to maintain optimal clinical net benefit in each specific context.

A DeLong test was performed to compare the discriminative ability between the two cohorts. No statistically significant difference was observed between the AUC values of the modeling cohort (G1) and the validation cohort (G2) (*Z* = 0.126, *P* = 0.899), indicating stable model performance across diverse populations.

### 3.6 Sensitivity and subgroup analyses

To evaluate the stability of the model in the absence of family history data, subgroup analyses stratified by sex were performed. The five independent predictors (age, BMI, smoking history, alcohol consumption, and high-fat diet) remained significantly associated with the risk of colon cancer and precancerous lesions in both male and female subgroups (all *P* < 0.05). Sensitivity analysis further demonstrated that adding sex to the multivariable model did not significantly improve the C-index (0.765 vs. 0.768, *P* = 0.42), confirming that the selected factors provide robust predictive value across different demographic profiles (Supplementary Figure 1).

## 4 Discussion

Colon precancerous lesions are closely linked to the development of colon cancer and may progress to malignancy through various carcinogenic pathways. Therefore, early detection and timely management of these lesions are of great significance for the primary prevention of colon cancer. Although colonoscopy remains the gold standard for the detection and diagnosis of colon cancer and its precursors, its invasive nature, associated risks, and requirement for extensive bowel preparation contribute to low patient adherence in population-based screening programs (18, 19). In this context, the present study systematically investigated the independent risk factors for colon cancer and precancerous lesions. Multivariable regression analysis identified age, BMI, smoking history, alcohol consumption, and high-fat diet as significant predictors associated with the development of colonic neoplasia. It is noteworthy that this study specifically targeted colonic rather than rectal lesions, based on evidence that lifestyle factors—such as high-fat diet and metabolic dysregulation—may exert differential etiological effects along the colorectal continuum, with stronger associations frequently observed for colon carcinogenesis.

Age is a well-established risk factor for both colon cancer and its precursors (20, 21). With advancing age, the efficiency of cellular DNA repair mechanisms progressively declines, while cumulative exposure to carcinogenic agents increases the likelihood of somatic mutations, thereby elevating the risk of colonic neoplasia (22). However, recent epidemiological studies have reported a decline in colon cancer incidence among older adults over the past several decades, accompanied by a concerning rise in early-onset colon cancer among individuals under 50 years of age (23). This trend is likely attributable to shifts in modern lifestyle patterns, particularly the increasing prevalence of sedentary behavior and high-calorie dietary habits. Furthermore, current screening guidelines predominantly target older populations, potentially leading to delayed diagnosis and advanced-stage presentation in younger individuals with early-onset disease (24). These observations underscore the urgent need for enhanced public health education and the promotion of healthy lifestyles—including regular physical activity and reduced caloric intake—to mitigate the risk of premature colon cancer.

Overweight, defined as BMI >25 kg/m², represents another modifiable risk factor that cannot be overlooked. Studies have demonstrated that obese individuals have a 1.56-fold higher risk of colon cancer compared to their normal-weight counterparts (25). A meta-analysis of 13 observational studies by Karahalios et al. (26) further revealed that each 5 kg increase in body weight was associated with a 3% increase in colon cancer risk. The underlying mechanisms linking excess adiposity to colon carcinogenesis are multifactorial. Adipose tissue in obese individuals secretes pro-inflammatory cytokines, such as tumor necrosis factor-alpha (TNF-α) and interleukin-6 (IL-6), which contribute to chronic low-grade inflammation (27, 28). Concurrently, obesity-related insulin resistance and oxidative stress may promote the proliferation and survival of colon cancer cells. Hyperinsulinemia, a common feature of obesity, can activate the insulin-like growth factor-1 (IGF-1) signaling pathway, further enhancing tumorigenic potential (29). Therefore, weight management and maintenance of a healthy BMI should be integral components of colon cancer prevention strategies.

Cigarette smoking significantly elevates the risk of colon cancer and precancerous lesions (30). Carcinogenic compounds in tobacco smoke, including polycyclic aromatic hydrocarbons and nitrosamines, may reach colonic mucosa via the bloodstream or direct ingestion, where they induce genetic mutations—particularly in oncogenes and tumor suppressor genes (31). Smoking also promotes oxidative stress and inhibits apoptosis, thereby facilitating the proliferation of neoplastic cells. A study by Botteri et al. (32) found that both current and former smokers exhibited an increased risk of colon cancer compared to never-smokers, with risk magnitude correlating positively with smoking intensity and duration. Importantly, smoking cessation has been shown to reduce colon cancer risk, underscoring the critical role of tobacco control in cancer prevention (33, 34).

Alcohol consumption is another well-documented risk factor for colon cancer, with its effects mediated through multiple biological pathways. These include the genotoxicity of acetaldehyde, tissue damage from ethanol metabolites, generation of reactive oxygen and nitrogen species, alterations in folate metabolism, and alcohol-induced dysbiosis of the gut microbiota (35, 36). Fang et al. (37) demonstrated that individuals consuming 15 g or more of alcohol daily had a significantly higher incidence of colon cancer compared to non-drinkers. This association is attributed to acetaldehyde accumulation, which can induce DNA damage and impair mismatch repair mechanisms, thereby increasing mutational burden. Furthermore, Hur et al. (38) reported that alcohol intake during early adulthood was positively associated with colon cancer risk in a dose-dependent manner, with stronger correlations observed in males. Drinking frequency also emerged as a significant determinant, with higher frequency conferring greater risk. Collectively, these findings highlight the importance of moderating alcohol intake to reduce the burden of colon cancer and its precursors.

Dietary factors, particularly high-fat diet, play a pivotal role in colon carcinogenesis (39). A large-scale prospective cohort study revealed that elevated consumption of saturated fatty acids is associated with increased colon cancer risk (40). Mechanistically, high-fat diet has been shown to alter the composition and diversity of the gut microbiota, promoting the expansion of pro-carcinogenic bacteria such as *Escherichia coli* and *Bacteroides fragilis*. The toxins and metabolites produced by these bacteria can damage the intestinal mucosa and drive tumor formation (41). Additionally, obesity and metabolic syndrome resulting from chronic high-fat dietary patterns may further compound colon cancer risk. Therefore, optimizing dietary habits—particularly reducing fat intake—is essential for effective colon cancer prevention (42).

This study successfully constructed a risk assessment model that offers a distinct clinical edge over previous tools through its streamlined focus on modifiable lifestyle factors; this provides a pragmatic, low-cost solution for rapid risk stratification where detailed medical or genetic histories are difficult to obtain; furthermore, while synergistic interactions between lifestyle factors—such as the combined impact of alcohol and tobacco use—are clinically recognized, they were excluded from this model to prioritize a streamlined scoring framework and avoid potential overfitting associated with increased model complexity. As a visual tool, the nomogram assigns specific points to each variable through an intuitive graphical display. These points are arranged in sequence along the lines on the chart to form a continuous curve. This method is not limited to a graphical representation, but is a more accurate means of predicting individual clinical events. In the modeling cohort, the calibration curve showed that the C-index of colon cancer and precancerous lesions was 0.763 (0.714-0.813), indicating that the nomogram prediction results were highly consistent with the actual observation results. The decision curve analysis results showed that when the risk threshold was >0.15, intervention based on the nomogram prediction results could provide more clinical net benefits. In the external validation, the validation cohort achieved a C-index of 0.761. ROC curve analysis indicated an AUC value of 0.761 for the model. The outcomes further indicated that the model developed in this study exhibited moderate discrimination and acceptable calibration, reflecting a balanced predictive performance suitable for preliminary screening. The striking stability of the AUC values (0.765 vs. 0.761) across these heterogeneous cohorts may initially appear anomalous; however, it likely reflects the fundamental and universal oncogenic impact of the selected modifiable risk factors—such as obesity and tobacco use—which consistently drive colorectal carcinogenesis regardless of genetic background. Nonetheless, we recognize that this near-perfect consistency could also mask potential overfitting to common lifestyle patterns, and the model’s “direct transport” without extensive recalibration of the intercept remains a caveat for its global application. The observed disparity in optimal risk thresholds (0.15 in the clinical cohort vs. 0.11 in NHANES) raises important considerations for clinical implementation; this shift reflects the lower baseline prevalence of self-reported lesions in the validation set and suggests that while the core predictors are stable, the intervention threshold must be locally calibrated to the specific epidemiology of the target population to maintain its clinical utility.

This study has several limitations. First, a significant phenotypic discrepancy exists between our cohorts: while the modeling group relied on precise pathological diagnosis, the NHANES validation group utilized the MCQ module, which is prone to self-report bias. Since ordinary respondents often accurately report a “cancer” diagnosis but frequently overlook or fail to recall specific “precancerous lesions” (such as asymptomatic adenomas) without a recent pathology report, the validation set may primarily represent established malignancy. This potentially shifts the target phenotype in the validation dataset, which may omit the very precancerous population the model aims to predict, thereby limiting the interpretability of our external validation results for early-stage screening. Second, validation based on the U.S. NHANES dataset may limit its applicability to the Chinese population. Third, the retrospective nature of the study design introduces potential selection bias. Fourth, the dichotomization of age and BMI at specific clinical thresholds (>55 years and >25 kg/m²), while enhancing the model’s practical utility for rapid screening, represents a methodological trade-off that may lead to a loss of information and a slight underestimation of the predictive power inherent in the raw continuous variables. Specifically, the absence of family history—a well-established independent risk factor—is a significant limitation. This omission may lead to an underestimation of individual risk for genetically predisposed populations, potentially reducing the model’s overall sensitivity. However, our sensitivity analysis demonstrates that modifiable lifestyle factors (BMI, smoking, and diet) remain strong, independent predictors regardless of demographic variations, suggesting that the model remains a valuable tool for identifying high-risk individuals based on lifestyle-related exposures. This is further supported by our subgroup analysis, which demonstrated that the predictive power of modifiable factors like BMI and diet remained consistent regardless of gender, mitigating concerns that the absence of genetic data might disproportionately skew the model’s performance in certain populations. Fifth, while the modeling cohort provided sufficient EPV, the validation cohort contained only 93 events, which may be insufficient to robustly validate a model intended for clinical screening decisions. This relatively small sample size may affect the accuracy of advanced metrics such as the net reclassification improvement (NRI). Sixth, the reliance on self-reported cancer history in the NHANES validation cohort means that the model’s ability to specifically predict precancerous lesions—rather than established malignancy—remains inadequately validated. The underreporting of asymptomatic adenomas may have biased the validation toward more advanced disease, limiting the model’s applicability for early screening. Future external validation should ideally include cohorts with histologically confirmed precancerous lesions to fully assess the model’s performance in the target population. Future studies require larger-scale, prospective, and multi-center datasets to further investigate the model’s intercept calibration across diverse populations and to ensure that the observed performance consistency is not an artifact of data-cleaning processes or localized lifestyle similarities. Furthermore, validating the model across diverse racial, socioeconomic, and geographic populations is essential to ensure its broad applicability. Unlike many validated models based on pre-pandemic cohorts, a unique clinical advantage of our nomogram is its validation using the 2021–2023 NHANES dataset; this ensures the tool is accurately calibrated to contemporary post-pandemic dietary patterns and lifestyle trends, making it more relevant for current clinical practice. This integration into population-based colorectal cancer screening programs remains a key objective for future research.

## 5 Conclusion

This study developed a nomogram-based predictive model for colon cancer and precancerous lesions using accessible lifestyle and clinical factors, including age, BMI, smoking, alcohol consumption, and high-fat diet. The model demonstrated moderate but consistent predictive performance in both internal and external validation cohorts. While these findings suggest potential utility in supporting risk-stratified colorectal cancer screening strategies, the model’s direct clinical applicability remains constrained by its retrospective design, limited sample size, uncertainty regarding optimal risk threshold stability across diverse populations, the omission of potential interaction effects among lifestyle predictors, and the inherent differences in outcome definitions between pathologically confirmed and self-reported cases. While the absence of genetic data may constrain the model’s comprehensive risk evaluation, our findings underscore the substantial impact of modifiable lifestyle factors, providing a pragmatic screening support tool for populations where detailed genetic histories are difficult to obtain. Therefore, larger-scale, prospective, multi-center studies incorporating additional clinical and molecular variables are warranted to further refine and validate the model prior to its routine clinical implementation.

## Data Availability

The raw data supporting the conclusions of this article will be made available by the authors, without undue reservation.
